# The Changes in the Inner-Structure and Mechanical Strength of the Composite Cement Materials and Silica-Carbon Nanotube-Nylon 66 Electrospun Nanofibers

**DOI:** 10.3390/polym16172475

**Published:** 2024-08-30

**Authors:** Tri N. M. Nguyen, Huy Q. Nguyen, Jung J. Kim

**Affiliations:** 1 Faculty of Civil Engineering, Industrial University of Ho Chi Minh City, 12 Nguyen Van Bao, Ward 4, Go Vap District, Ho Chi Minh City 700000, Vietnam; nguyennhatminhtri@iuh.edu.vn; 2 Department of Civil Engineering, Kyungnam University, 7 Kyungnamdaehakro, Masan, Changwon-si 51767, Republic of Korea; nguyenquochuy@muce.edu.vn

**Keywords:** nylon 66, nanosilica, carbon nanotubes, electrospun nanofibers, cement, mechanical strength, microstructure

## Abstract

This study presents the feasibility of improving some selected mechanical strengths and the inner-structural analyses of cement matrix by electrospun nanofibers containing nylon 66, nanosilica, and carbon nanotube. The hybrid electrospun nanofibers were fabricated and mixed into ordinary Portland cement. From the mechanical strength test results, the hybrid nanofibers have shown their role in improving the tensile, compressive, and toughness behavior of the mixed cement material. The improvements of 62%, 38%, and 69%, respectively, were observed compared to those of the control paste. The novelty of the surface and inner structure of the hybrid fibers, as well as the modified cement matrix, were observed by the scanned images from electron microscopes. Besides, the additional pozzolanic reaction between the generated calcium hydroxide and the attached silica was clarified thanks to the results of energy dispersive spectroscopy, X-ray diffraction, and thermal gravimetric analysis. Finally, the consistency between mechanical strength results and inner-structure analyses showed the potential of the proposed fiber to improve cement-based materials.

## 1. Introduction

Cement is a principal component of concrete, the most popular building material worldwide [1]. From the cement strength perspective, silica has emerged as a pivotal additive in recent years. Costa et al.’s finding showed the effective amount of silica on cement compressive behavior in high-temperature conditions [2]. The study of Lavergne et al. reported the influence of silica particles in nanosize on some characteristics of cement paste, such as accelerating the hydration process but changing the cement paste workability and promoting the early age strength of the cement paste [3]. Besides, the role of silica fume when it is added to Portland cement in promoting the compressive strength and durability of concrete under the sulfuric acid environment was presented in [4]. Based on the study of Garg et al., the development of microstructure and enhancement of strength of cement matrix when adding pozzolanic admixtures like nanosilica was reported [5]. Recently, a study [6] denoted the effects of adding a small amount of silica fume on reducing water demand, water absorption, and exothermic heat of hydration, improving the later strength mechanical properties, consuming calcium hydroxide, and creating an extra calcium silicate hydrate in the cement-fly ash system.

Alternatively, discovered in 1991 [7], carbon nanotubes (CNTs) have proven their ability in materials science, especially as a useful additive for enhancing cement characteristics. Adequate evidence indicates that the bridging and filling effect of CNTs inside the cement matrix improves matrix strength. Upon adding 0.1 wt% multi-walled carbon nanotubes (MWCNTs), an increase of 15% in the 28-day compressive strength of the cement paste was observed [8]. Zhang et al. [9] reported an improvement of 15.8% in flexural strength with 0.3% MWCNTs and polycarboxylate superplasticizer dispersants. In addition, Şimşek 2020 [10] reported an increase of 77.2% in the splitting tensile strength of the cement paste with 0.1 wt% MWCNTs. Although NS and CNTs have demonstrated their ability to improve the mechanical characteristics of cementitious materials, these supplementary materials display an agglomeration phenomenon in aqueous solutions, resulting in adverse effects of reduced strength and elastic modulus of the cement-based materials. It has been shown that agglomeration occurs because of the high specific surface area and high surface energy of these nano-sized materials [11,12,13,14]. However, numerous studies have solved this issue using surfactants [9,15], surface treatment [16,17,18], and ultra-sonication energy [19,20,21].

Previous works showed the effect of electrospun nanofibers on some selected mechanical characteristics of ordinary Portland cement (OPC) [22,23,24,25]. An indirect way to improve the tensile behavior of OPC by CNTs was shown in [22], where CNTs were first introduced into electrospun nylon 66 nanofibers, followed by blending the as-spun nanofibers into cement. The bridging effect of the unmodified and CNT-modified nylon 66 nanofibers inside the matrix, together with the increase in tensile strength, proved the feasibility of this proposed method to improve cement material. It is necessary to provide insights into problems and develop potential research ideas or hypotheses on improving cement strength by electrospun nanofibers. Therefore, the present work concentrates on the effect of a hybrid version of electrospun nylon 66 nanofibers containing nanosilica and carbon nanotubes (NS-CNT-N66 NFs) on cement’s mechanical and microstructural perspectives. The fibers were fabricated and mixed into the cement powder with the help of an improved collector. Mechanical strength tests were conducted to examine the tensile and compressive behavior of OPC. In addition, scanning electron microscopy (SEM), transmission electron microscopy (TEM), energy dispersive spectroscopy (EDS), X-ray diffraction (XRD), and thermal gravimetric analysis (TGA) were conducted to clarify the change in the microstructure of the fibers and the modified cement matrix.

## 2. Experiments

### 2.1. Polymer Solution

**Figure 1:**
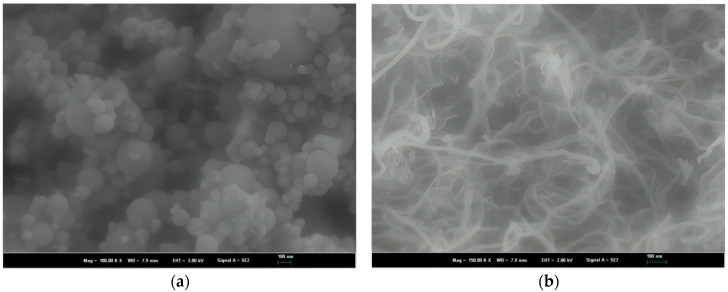
Morphology of materials: (**a**) silica particles and (**b**) CNT.

**Figure 2:**
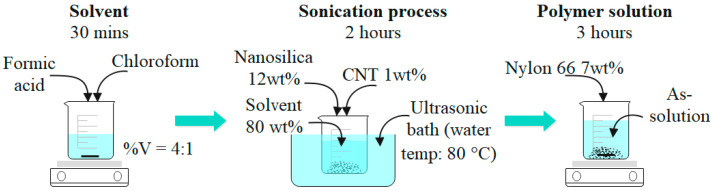
Polymer solution preparation.

The precursors of polymer solution are nylon 66 (N66, Sigma-Aldrich, USA), nanosilica (NS, Elkem Microsilica, Norway), and carbon nanotube (CNT, Hanos, Korea). Table 1, Table 2 and Table 3 show some characteristics of the precursors. Figure 1 shows the morphology of nanosilica and carbon nanotubes. The solvent is a mix of formic acid and chloroform (Daejung–Korea) with a volume ratio of 4:1. The polymer solution preparation was conducted following the schematic in Figure 2. The precursors’ weight ratio was N66—7 wt%:NS—12 wt%:CNT—1 wt%. This ratio was chosen under the trial-error procedures, finally observing the polymer solution with suitable viscosity for the electrospinning process. The stirring of solvent, sonication process, and mixing polymer solution were conducted in 30 min, two hours, and three hours, respectively. The sonication process was conducted during polymer solution preparation to avoid the precipitation phenomenon due to the van der Waals forces between the nanomaterial molecules and to obtain a homogeneous solution [19,20,21]. The as-polymer solution was kept for 24 h under laboratory conditions to relax the polymer chain before the nanofiber fabrication process.

### 2.2. Electrospinning Process, Nanofiber Blended Cement Composite

**Figure 3:**
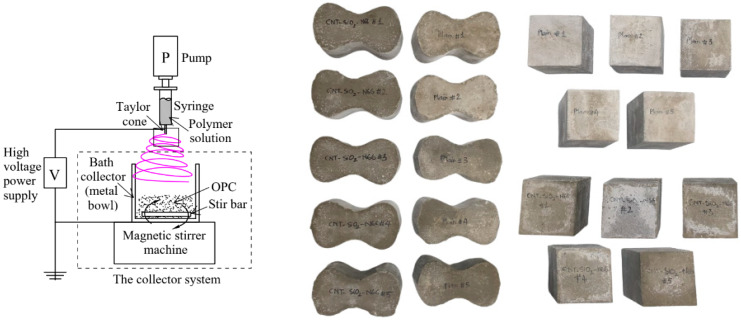
Electrospinning and blending process, samples for tensile strength and compressive strength test.

The electrospinning system fabricated nanofibers. The input parameters of the electrospinning process are referred to in Table 4. The nanofibers were spun directly into an improved collector containing a metal bowl, magnetic stir bar, and a magnetic stirrer machine supplying rotated energy for blending nanofibers and cement particles (see Figure 3). Cement type I [26] (Ssangyong—Korea), 95 wt%, was prepared in a metal bowl. The cement’s properties can be referred to in Table 5. The nanofiber ratio was chosen at 5 wt%. Due to the variation in the microstructure of the cement paste and the difficulty of examining the spontaneous cracking that occurs during the curing period, it is necessary to conduct experiments with small specimens. According to ASTM C307 and C109/109 M, for examining the tensile and compressive behavior of hydraulic cement mortar [27,28], five dog bond specimens and five cubic specimens were prepared with a water-binder ratio of 0.5 [29] (see Figure 3). The specimens were tested after 28 days of curing in water at a room temperature of 23 ± 2 °C and with a relative humidity of 50%.

### 2.3. Testing Methods

In this study, the tensile strength was observed by the mortar tensile strength test apparatus with a capacity of 5 kN. The compressive strength was observed by the hydraulic universal testing machine with a capacity of 1000 kN. ASTM standards C307 and C109/109M were referred to conduct the above mechanical tests, respectively [27,28]. SEM, TEM, and EDS analyses were conducted to analyze the morphology of nanofibers, the microstructure of hardened cement pastes, and the local chemical components of the failure specimens. SEM and EDS were done using the Zeiss Merlin Compact system. The input parameters were set up as the accelerating voltage of 5 kV and 12 kV, respectively; the working distance was 9 mm. The samples were coated with a 5Å-platinum layer to get the highest resolution. The TEM was done by the FEI Tecnai F30 Twin system under the acceleration voltage of 300 kV. Besides, the XRD analysis was conducted by the D8 Advance system under the input parameters of the Cu kα radiation (40 kV, 40 mA), the scanning speed of 0.4 s/step, and the step size of 0.02° (2θ)/step from 5° to 70°. Finally, the thermal analysis was conducted by TA instrument SDT-Q600, with the input parameters as the heating range from room temperature to 1000 °C under the Nitrogen atmosphere, the flow rate of 100 mL/min, and the heating velocity of 10 °C/min.

## 3. Results and Discussion

### 3.1. Mechanical Strength

**Figure 4:**
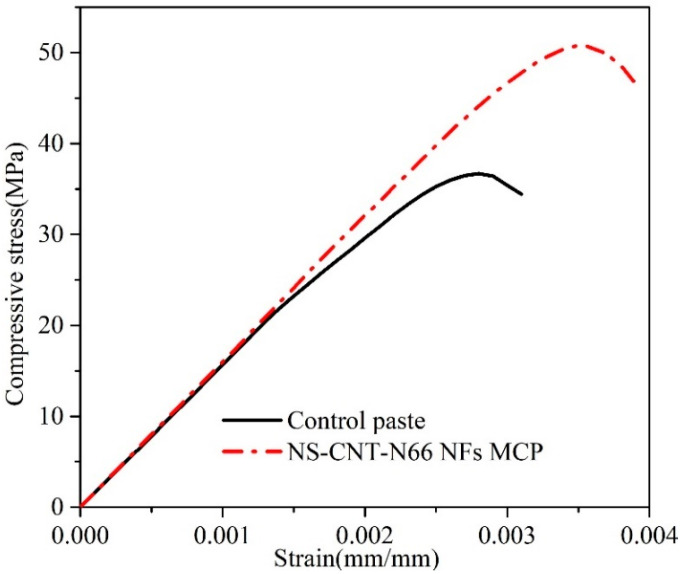
Constitutive curves of the hardened cement pastes.

The influence of the hybrid version of electrospun nylon 66 nanofibers containing nanosilica and carbon nanotubes on the mechanical characteristics of hardened cement pastes has been estimated through the 28-day tensile and compressive strength tests. The observations in Figure 4 and Table 6 show the efficiency of NS-CNT-N66 NFs in increasing the compressive behavior of cementitious materials. The increases of 38% and 69% in compressive strength and toughness were observed, respectively, compared to those of the control paste. Note that the toughness of the material was calculated according to Timoshenko and Gere’s theory as equal to the area limited by the constitutive curves and strain axis [30]. As published earlier, the work [22] found that the compressive strength of cement paste modified by electrospun nylon 66 nanofibers containing CNTs increased by 14% compared to that of the control paste, while there was no change in toughness when modifying cement by this version of e-spun nylon 66 NFs. The difference between the versions of e-spun nylon 66 NFs in this present study and the previous study [22] is that the silica particles have been proposed to attach alongside the fibers. The reason for proposing the silica particles is thanks to the extra pozzolanic reaction or calcium hydroxide (CH) consumption of silica during the cement hydration period, which is found in the literature [3,5,6]. Based on this assumption, we conducted microstructural analyses of hardened cement pastes to figure out whether the change in the cement matrix may meet with the change in compressive behavior of the NS-CNT-N66 NFs modified cement paste. From a tensile strength perspective, the result in Table 6 shows an increase of 62% compared to that of the control paste. In the previous study, we reported an increase of 57% in the tensile strength of the cement paste modified by electrospun nylon 66 nanofibers containing CNTs [22]. Notably, the first time the role of CNTs in reinforcing inside the e-spun nylon 66 was found, then this hybrid version of e-spun nanofibers showed effectiveness in increasing the tensile strength of cement. In this present study, due to the appearance of silica particles inside the cement matrix by attaching alongside fibers, tensile strength showed a better result compared to that reported in the literature. Similar to the above assumption, a change in the inner structure of the cement matrix may occur due to the extra chemical reaction during the hydrate period. Better linking between fibers and cement hydration products may be generated. Hence, this leads to an increase in the tensile behavior of the modified cement paste. The next sections will discuss the analysis of the cement matrix’s microstructures.

### 3.2. Morphology and Microstructure of Nanofibers

**Figure 5:**
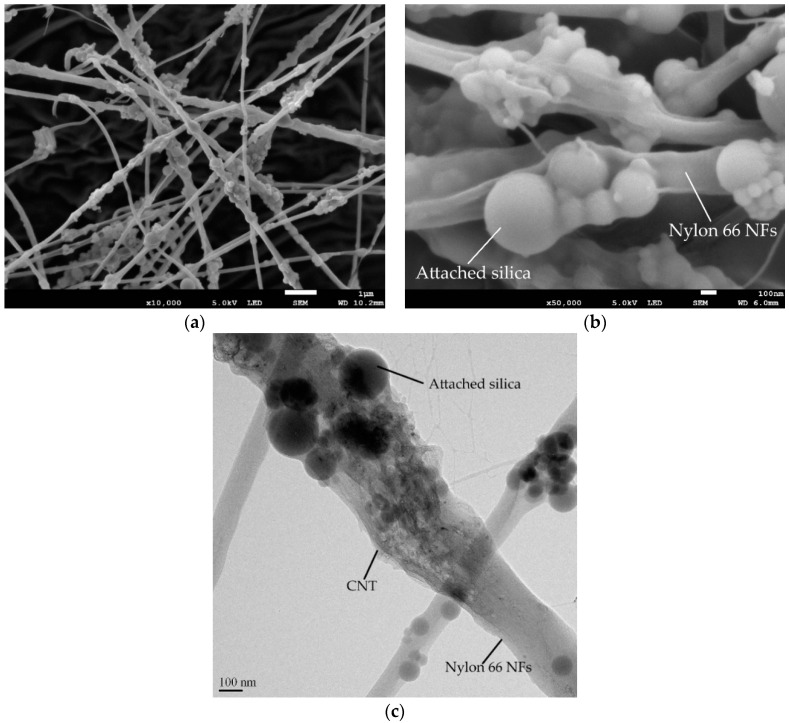
Microstructure of the electrospun nanofibers: (**a**,**b**) SEM images of the fibers under low and high magnifications, (**c**) TEM image of the fibers.

Figure 5 depicts the morphologies of NS-CNT-N66 NFs in this study. In general, the electrospun nanofibers tend to overlap each other, forming net layers. As in Figure 5a,b, the bead-on-string morphology of nanofibers was shown, which was glossy and smooth. With this surface texture, it seems to be good to incorporate these nanofibers into cement paste due to the numerous connecting points between the surface of nanofibers and cement hydration products. The mean diameters were estimated at 170 nm, and the bead size was specified from 40 to 570 nm, consistent with the diameter of silica particles shown in Figure 1a. Under the TEM image that was shown in Figure 5c, CNTs were observed as disorganized forms, gathered around the silica beads and distributed along the fiber axis. The presence of the CNTs strengthened the nanofibers; as a result, the hardened cement pastes containing CNTs showed a better performance compared to that of the cement paste blended with N66 NFs [22,23].

### 3.3. Inner-Structure of Hardened Cement Pastes

**Figure 6:**
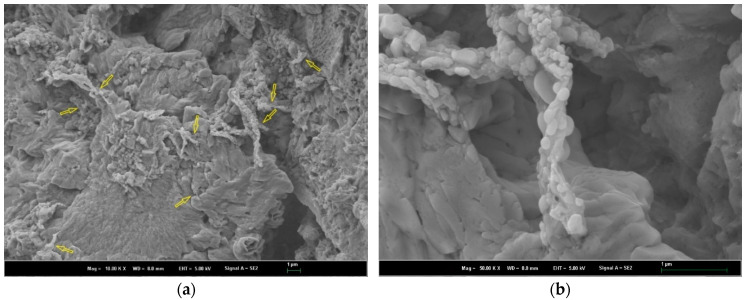
Microstructure of the cement matrix modified by NS-CNT-N66 NFs under low and high magnifications (the arrows show the location of the deformed fiber inside the cement matrix): (**a**) 10k-magnification; (**b**) 50k-magnification.

Figure 6 presents the microstructure of the hardened cement pastes blended with NS-CNT-N66 NFs. In this case, the bridging effect of nanofibers among the cement hydration products was observed. It is worth mentioning that, by the same method to incorporate nanofibers into the cementitious materials, the bridging effect of nanofibers inside the microstructure of the hardened cement pastes was also found [22,23,24]. Therefore, the method proposed in previous works has effectively incorporated electrospun nanofibers into cementitious materials. In this present study, in spite of the bridging effect that was observed in the microstructure of hardened cement pastes, it is difficult to realize the fibers inside the cement matrix due to the deformation of fibers. They were hidden among the hydration products. This phenomenon showed the role of attached silica particles in generating the extra pozzolanic reaction with CH created from the hydration process. The result of the extra pozzolanic reaction generated more calcium silicate hydrates (CSH) and consumed the created CH [3,5,6]. Hence, the morphology of nanofibers is deformed and well adhesive with cement hydration products such as calcium silicate hydrates (CSH), calcium hydroxide (CH), ettringite, and so on. As a result, the microstructure of the hardened cement pastes is more compacted. It is worth mentioning that CSH is known as the major hydration product of cement, which generates 50–60 percent of the volume of solids in a completely hydrated paste and provides the majority of the long-term strength and durability [31]. Therefore, the tensile and compressive strength of these modified pastes was increased compared to the findings from the previous study, which used nylon 66 nanofiber containing CNTs but without silica particles.

### 3.4. EDS Analysis

**Figure 7:**
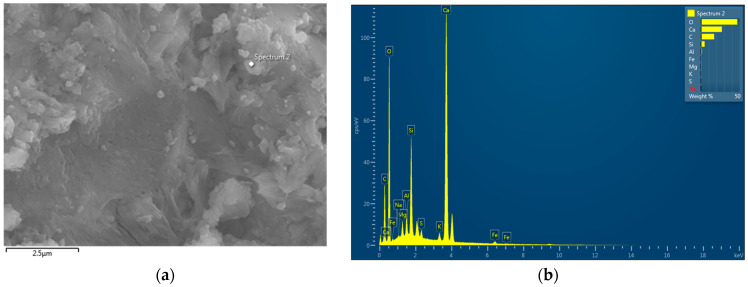

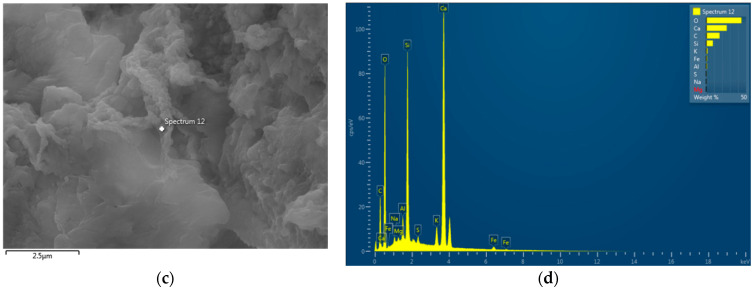
EDS analysis: (**a**,**b**) EDS spectrum of control cement paste; (**c**,**d**) EDS spectrum of NS-CNT-NFs MCP.

Figure 7 presents the EDS analyses of the control paste and the paste blended with NS-CNT-N66 NFs. In general, the main components observed in the EDS results of the two samples were consistent. A presence of Ca, Si, Al, and O, which are the main components of the cement hydration products, such as CH, CSH, and calcium aluminate hydrate (CAH) [1,31,32], was seen. Nevertheless, the amount of Si in the modified pastes was higher than that in the control paste, that is, 5.69% in the paste containing NS-CNT-N66 NFs compared to 3.17% in the control paste. These results explain the additional amounts of Si in the cement pastes from the electrospun nanofibers. In addition, a lower Ca/Si ratio has been observed with a higher supply of reactive silica for the pozzolanic reaction [33,34]. The Ca/Si ratio followed this order: paste blended with NS- CNT-N66 NFs < control paste (Table 7). Therefore, the supply of reactive silica for the formation of CSH was clarified.

### 3.5. XRD Analysis

**Figure 8:**
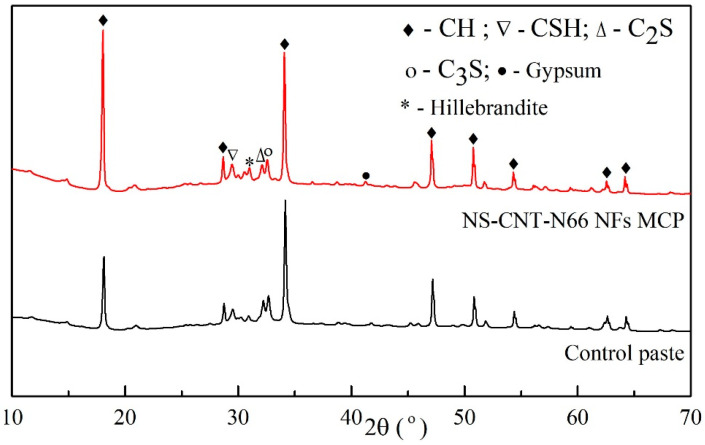
XRD patterns of the hardened cement pastes.

**Figure 9:**
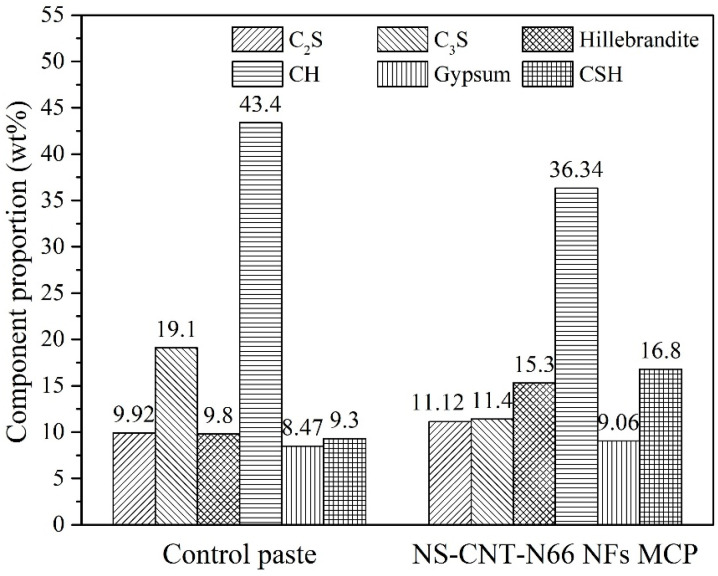
Component proportions of the cement matrices observed from the XRD results.

Figure 8 describes the XRD analysis results of the NS-CNT-N66 NFs-modified cement paste and the control paste. The XRD patterns were refined according to the Rietveld method [35], and the components in the cement matrix were determined according to the component structures of the hydration products, as shown in Figure 9. In general, no new products have been formed after the hydration process. Common hydration products such as CH, CSH, or some un-hydrated components such as Alite (C_3_S) and Belite (C_2_S) are recognized from the XRD patterns of all specimens. The XRD pattern of the control paste in this study is consistent with the findings of Jiang et al. [36]. Therefore, it can be used as the reference pattern in this present study. As can be compared with the literature, the CH component can be recognized by the peaks at 2θ of 18.1°, 28.7°, 34.1°, 47.1°, 50.8°, and 54.3°; CSH can be recognized by the peak at 2θ of 29.4°; C_2_S and C_3_S can be recognized by the peaks at 2θ of 32.3° and 32.67°, respectively. Figure 9 presents the ratio of components, which were calculated by the Profex program after refining the XRD patterns. According to this observation, the amount of CH decreased in contrast with the increase in the amount of CSH inside the modified cement matrix compared to that of the control paste. This can be a clear explanation for the assumption that the extra CSH was created inside the modified paste mentioned above [36,37]. It is worth noting that the amount of C_3_S in the paste containing NS-CNT-N66 NFs was lower than that in the control paste. Therefore, the higher increase in the CSH amount in this paste might also belong partly to the hydration of C_3_S. Based on the decrease-increase tendency of the CH and CSH amount, the consumption of CH due to the existence of attached silica is clarified. Therefore, the CSH amount increased, compacting the microstructure of the cement matrix [31]. Finally, the compressive behavior of the cement paste should be improved.

### 3.6. Thermal Analysis

**Figure 10:**
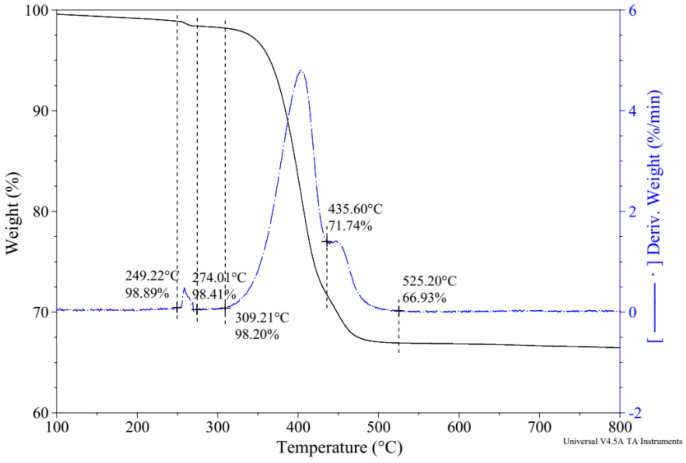
TGA-DTG results of the electrospun nanofiber.

The TGA-derivative thermogravimetry (DTG) results of the NS-CNT-N66 NFs are shown in Figure 10. Compared to the findings of Das et al. [38] and previous studies [22,23], the temperature range and phase detection can be referred to in Table 8. In addition, there was an incomplete decomposition process above 500 °C; that is, the silanol condensation was not complete at temperatures higher than 800 °C, which is in agreement with the results from Das et al. [38]. Based on this result, the decomposition range of NS-CNT-N66 NFs can be detected from 310 °C to 525 °C.

**Figure 11:**
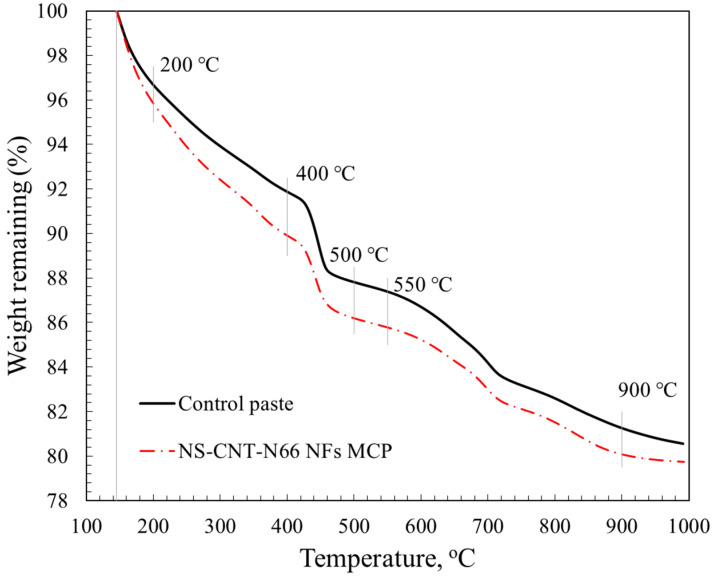
TGA curves of the hardened cement pastes.

Figure 11 shows the results from thermogravimetric analyses of the hardened control cement paste and the hardened cement pastes blended with NS-CNT-N66 NFs. Generally, the TGA results show the common curves for hardened cement paste. To the authors’ knowledge, the phases in the cement matrix can be detected by the TGA result as follows (Table 9):

As can be observed from the TGA results in Figure 11 and Table 10, the amount of CSH from the paste modified by NS-CNT-N66 NFs was higher than that of the control paste, 4.19% compared to 3.36%. The amount of CH was lower, 3.72%, compared to 4.07%, respectively. According to the above analysis, the decomposition temperature range of the CH phase and e-spun nanofiber overlaps from 400 °C to 500 °C; the amount of CH from the modified cement matrix still decreased compared to that of the control cement matrix (See Table 10). This observation shows suitably with the XRD results, the decrease-increase tendency of the CH and CSH amounts when adding NS-CNT-N66 NFs to cement. Therefore, the attached silica particles on the nylon 66 nanofibers affected the final product of the cement paste and confirmed the initial assumption regarding the role of the pozzolanic agent in the attached silica particles.

## 4. Conclusions

In this study, the influence of the hybrid electrospun nanofibers containing nanosilica, carbon nanotube, and nylon 66 on the inner structure and mechanical behavior of cement-based materials has been estimated. The following conclusions can be drawn according to the test results:

- There are increases of 38% and 69% in compressive strength and toughness, respectively, and an increase of 62% in tensile strength when incorporating the proposed nanofibers into the cementitious materials.
- The bead-on-string morphology of the hybrid nanofiber with the attached silica particles and the existence of CNTs inside the nanofiber are observed.
- The bridging and filling effect has been found by electron microscope analysis. The increase of CSH, thanks to the extra pozzolanic reaction, and the compacted structure of the modified cement matrix have been estimated through the EDS, XRD, and TGA analyses, explaining the mechanism of improvement in the strength of the composite cement-nanofiber materials.

Above all, the methodology for incorporating electrospun nanofibers into the cement matrix and the proposed nanofiber containing silica, carbon nanotube, and nylon 66 have been considered as a good approach to strengthening cement-based materials. However, for practical application, more work should be conducted, concentrating on the cement matrix characteristics, especially its behavior inside cement concrete.

## Figures and Tables

**Table 1:** Properties of Nylon 66.

Linear Formula	Molecular Weight (g/mol)	Abrasion Resistance (mg)	Autoignition Temp (°C)	Hardness	mp (°C (lit.))	Tm (°C)	Density at 25 °C (g/mL)
[-CO(CH_2_)_4_CONH(CH_2_)_6_NH-]n	262.35	7	400	121	250–260	270	1.14

**Table 2:** Properties of nanosilica.

Properties	SiO_2_ (%)	H_2_O (%)	LOI (%)	Coarse Particles > 45 μm (%)	Bulk Density (kg/m^3^)
Value	<90	<1.0	<3.0	<1.5	200–350

**Table 3:** Properties of CNT.

Properties	Purity (%)	Outer Diameter (nm)	Length (μm)
Value	93–97	9–10	10–30

**Table 4:** Input parameter for electrospinning process.

Properties	Voltage (kV)	Syringe (mL)	Needle’s Inner Diameter (mm)	Needle’s Outer Diameter (mm)	Needle Collector Distance (mm)	Pump Speed (μL/min)
Value	12	12	0.6	0.9	30	30

**Table 5:** Chemical composition and physical properties of the cement.

CaO	61.33
Al_2_O_3_	6.40
SiO_2_	21.01
SO_3_	2.30
MgO	3.02
Fe_2_O_3_	3.12
Ig. loss	1.40
Specific surface area (cm^2^/g)	2800
Compressive strength, 28-day (MPa)	36

**Table 6:** Tensile and compressive behaviors of the hardened cement pastes.

	Control Paste	NS-CNT-N66 NFs MCP
Compressive strength (MPa)	36.92 (1.1995)	50.91 (2.477)
Toughness (J/m^3^)	68,608.5	116,006
Tensile strength (MPa)	1.18 (0.0753)	1.92 (0.1602)

Where MCP is modified cement paste, the values in parentheses are standard deviations.

**Table 7:** Atomic ratio (%) of Ca and Si in the plain and modified pastes.

	Control Paste	NS-CNT-N66 NFs MCP
Ca	12.65	12.31
Si	3.17	5.69

**Table 8:** Observation from TGA-DTG of NS-CNT-N66 NFs.

Temperature Range (°C)	Phase Detection	Explanation
250 °C to 270 °C	Silica	The evaporation of the adsorbed water from the nanosilica surface [38]
310 °C to 480 °C	Nylon 66	[22,23]
435 °C to 525 °C	CNT	[22]

**Table 9:** Observation from TGA-DTG of cement paste.

Temperature Range (°C)	Phase Detection	Explanation
<145 °C	Arbitrary water	The evaporation of arbitrary water inside the matrix when conducting the thermal analysis under nitrogen and free carbon dioxide conditions [32]
145 °C to 200 °C	CSH	The dehydration of CSH [32,39,40,41]
400 °C to 500 °C	CH	The dehydration of CH [32,39,40,41]
550 °C to 900 °C	CaCO_3_	The decarbonation of calcite [32,39,40,41]

**Table 10:** The weight loss (%) from thermal analysis.

Temp (°C)	Control Paste	NS-CNT-N66 NFs MCP
145~200	3.36	4.19
400~500	4.07	3.72
550~900	6.14	5.71

## Data Availability

The original contributions presented in the study are included in the article, further inquiries can be directed to the corresponding author.
